# Clinical relevance of different WHO grade 3 pancreatic neuroendocrine neoplasms based on morphology

**DOI:** 10.1530/EC-17-0388

**Published:** 2018-01-30

**Authors:** Xu Han, Xuefeng Xu, Hongyun Ma, Yuan Ji, Dansong Wang, Tiantao Kuang, Wenchuan Wu, Bin Song, Gang Li, Gang Jin, Wenhui Lou

**Affiliations:** 1 Department of Pancreatic SurgeryZhongshan Hospital, Fudan University, Shanghai, China; 2 Department of Pancreatic SurgeryChanghai Hospital, Second Military Medical University, Shanghai, China; 3 Department of PathologyZhongshan Hospital, Fudan University, Shanghai, China

**Keywords:** pancreatic neuroendocrine neoplasms, grade 3, well-differentiated pancreatic neuroendocrine tumors, poorly differentiated pancreatic neuroendocrine carcinomas, prognosis

## Abstract

**Purpose:**

Emerging evidence suggests G3 pancreatic neuroendocrine neoplasms (pNENs) present heterogeneous morphology and biology. The 2017 WHO classification has introduced a new category of well-differentiated pancreatic neuroendocrine tumors (WD-pNETs) G3, compared with poorly differentiated pancreatic neuroendocrine carcinomas (PD-pNECs) G3. We aim to analysis the demographics and outcomes of patients with resectable 2017 WHO G3 pNENs to facilitate the distinction between two entities.

**Methods:**

The multi-institutional retrospective cohort involving 57 surgically treated patients affected by 2017 WHO G3 pNENs were morphologically identified and clinically analyzed. Patients having WD-pNETs G3 and those having PD-pNECs G3 were compared.

**Results:**

Thirty patients had WD-pNETs and 27 patients had PD-pNECs. The distributions of Ki-67 and mitotic count in patients with PD-pNECs or WD-pNETs showed remarkable disparities. ROC indicated cut-off value of Ki-67 was 45. PD-pNECs were more common in patients with elevated Ki-67 and mitotic count, advanced AJCC TNM stage, vascular invasion, regional lymph-node metastases, elevated NSE and decreased CgA levels compared with WD-pNETs (*P* < 0.05). The association between 2017 WHO G3 grade and TTR was statistically significant (*P* < 0.05). Univariate analysis indicated OS rates were associated with morphologic differentiation (WD-pNETs vs PD-pNECs), Ki-67, TNM staging, synchronous distant metastases, initial treatments, vascular invasion, regional lymph nodes metastases, mitotic count and age (*P* < 0.05). Multivariate analyses illustrated Ki-67, differentiation, TNM staging and vascular invasion were independent predictors (*P* < 0.05).

**Conclusions:**

PD-pNECs G3 presented malignant biological behavior and dismal outcome compared with WD-pNETs G3. These findings challenge 2010 WHO classification and suggest the categorization can be improved by refined tumor grading.

## Introduction

Pancreatic neuroendocrine neoplasms (pNENs) are rare and heterogeneous neoplasms of the pancreas, which make up 1–5% of all pancreatic neoplasms (1, 2). The incidence and prevalence of pNENs have been increasing significantly over the last decades (3, 4). The pNENs present in diverse tumor biology and manifest a wide spectrum of clinical behavior with varying degrees of malignancy.

The 2010 World Health Organization (WHO) classification utilized tumor proliferative ability (both Ki-67 proliferation index and mitotic rate) to divide pNENs into 3 pathological grades: low grade (G1), intermediate grade (G2) and high-grade (G3). According to 2010 WHO classification scheme, high-grade pNENs are defined as a tumor with Ki-67 proliferation index greater than 20% or mitotic rate greater than 20 per 10 high power fields (5). Traditionally, G3 pNENs have been recognized as poorly differentiated pancreatic neuroendocrine carcinomas (PD-pNECs), which further subdivided into small cell and large cell subtype, and were associated with highly aggressive behavior and dismal prognosis (6). However, recent literature indicates that some G3 pNENs containing noticeably different genetic mutational profile have well-differentiated morphology and a distinctly favorable outcome (7, 8). These G3 pNENs were also known as highly proliferative group of WD-pNETs (9). The clinical outcome and therapeutic strategy of WD-pNETs are also notably different from those of PD-pNECs (10).

It is believed that tumor grade may reflect inherent tumor aggressiveness. However, emerging evidence suggests that the 2010 WHO G3 category may not sufficiently distinguish highly proliferative WD-pNETs from PD-pNECs due to the lack of well-defined histologic criteria (11). The well-differentiated and poorly differentiated groups of pNENs have genetically distinct etiologies (12). Besides, current National Comprehensive Cancer Network (NCCN) guidelines recommend platinum-based chemotherapy as the primary treatment for both G3 highly proliferative WD-pNETs and PD-pNECs; however, evolving studies suggested that highly proliferative WD-pNETs with intermediate Ki-67 index may not respond as well to platinum-based chemotherapy as patients with PD-pNECs or extremely high Ki-67 index (10), reflected the existence of a grade-discordant group of high-grade pNENs with unique clinical characteristics and the correlation between the Ki-67 index and effect of platinum-based chemotherapy (13). Furthermore, the role of surgery in the treatment of patients with G3 pNENs remains obscure (14), and it is unknown whether surgery-based treatments for G3 pNENs could achieve a better survival benefit compared with chemotherapy-based treatments. Thus, patients with G3 pNENs are morphologically and biologically heterogeneous.

As suggested earlier, pNETs and pNECs may overlap in their proliferation index, making the distinction between them difficult and leading to therapeutic uncertainties. Recently, the 2017 WHO classification of pNENs has introduced a new category of WD-pNETs G3, defined by a Ki67 index greater than 20%, to the existing pNEN categories, which was helpful for the distinction of WD-pNETs G3 from PD-pNECs (15, 16). The 2017 WHO classification depicts the main differences in the morphological appearances of the pNENs and their variable proliferative activity. However, due to the rarity of G3 pNENs and tumor heterogeneity, there are few data available from large multicenter for Chinese population. Consequently, it is important to clarify the clinicopathological characteristics of G3 pNENs patients and provide much-needed validation of effectiveness of the 2017 WHO classification. The purpose of this study was to analyze the clinical features and outcomes of patients with resectable heterogeneous G3 pNENs in Chinese population from two large centers’ long-term updated experience to facilitate the distinction between two fundamentally different groups of pNENs. This study includes only primary G3 pNENs and excludes MiNEN to maintain a homogeneous population.

## Materials and methods

### Patient selection

The study protocol was approved by the medical ethical committees of Zhongshan and Changhai hospital who waived the need for patient consent for this study when individual patient consents were not identified. All 57 patients with G3 pNENs were enrolled from two independent institutions. We recruited consecutive 32 patients with G3 pNENs underwent surgery from Jan 2008 to Dec 2016 in Zhongshan Hospital, Fudan University and 25 patients with G3 pNENs underwent surgery in Changhai hospital, Second Military Medical University from Jan 2006 to Dec 2014. Clinical and demographic data were collected by medical record review in a standardized manner using predefined definitions. The eligibility criteria were as follows: all patients were potentially resectable at baseline; patients had an exact morphological diagnosis of G3 pNENs with specific Ki-67 proliferation index and mitotic rate in primary and/or recurrent/metastatic lesions, as well as well-defined histologic differentiation. Patients were excluded if any of following conditions were present: mixed neuroendocrine–non-neuroendocrine neoplasm (MiNEN); incomplete medical records.

In our series, pathological diagnostic criteria were mainly based on morphology and immunohistochemical assessment, through surgical specimen and intraoperative biopsy by institutional expert pathologists. The Ki-67 proliferative index was expressed as the percentage of Ki-67-positive cells in 2000 tumor cells within areas of highest immunostaining using the MIB1 antibody (Dako). Mitotic count was based on counting 50 HPF and in the area of highest mitotic activity and reported as number of mitoses per 10 HPF. In particular, morphologic differentiation refers to the extent of resemblance to the normal cellular counterpart. The diagnosis of nonfunctional tumors refers exclusively to tumors without clinical symptoms of excess specific hormones. In the case of multifocality, the largest lesion size was recorded. Measurement of serum CgA was performed using an enzyme-linked immunosorbent assay kit (CisBio Bioassays, Codolet, France), and serum NSE was measured using an electrochemiluminescence immunoassay automatic analyzer (Roche).

### Follow-up

The up-to-date follow-ups were usually repeated within 6 months or at appropriate shorter intervals on the basis of clinical conditions or when tumor relapse or metastasis was suspected. The duration of overall survival (OS) was calculated from the date of operation until tumor-specific death or the patient’s last follow-up. The time-to-relapse (TTR) duration was computed from date of remission of R0 resection to recurrence or metastases. The median follow-up time was 2.21 years (range, 0.20–9.12 years).

### Statistical analysis

The statistical analyses were performed using the SPSS statistical package version (16.0; SPSS). Student *t* test and Mann–Whitney *U* test were used to compare means when appropriate, whereas Pearson *χ* ^2^ test, Fisher exact test and Pearson correlation test were used to compare proportions when appropriate. Data of Ki-67 and mitotic count were used to construct the receiver operating characteristic (ROC) curve. The area under the ROC curve indicates the capability of indicators to discriminate between WD-pNETs and PD-pNECs. Survival was estimated according to the Kaplan–Meier product limit method and Life Tables method. The log-rank test was used to compare survival curves between different groups. Multivariate analyses using the Cox proportional hazards model were carried out to identify factors independently associated with prognosis. Risk factors were expressed as the hazard ratio (HR, 95% confidence interval (CI)). Statistical significance was defined as *P* < 0.05.

## Results

### General data

Among 57 pNENs patients with 2017 WHO G3 grade, 28 patients were men and 29 were women. The median age at diagnosis was 54 years (range, 21–82 years), and the mean (s.d.) age was 52.8 (13.3) years. The median size of the tumor (in the case of multifocality, the largest lesion was recorded) was 3.6 cm (mean (s.d.) size, 4.8 (3.0) cm; range, 1.8–17 cm). In 3 cases, the tumor was multifocal. The majority of G3 pNENs were located in the distal part of the pancreas, followed by the head. Regarding to morphologic differentiation, thirty patients were WD-pNETs G3, whereas 27 patients were PD-pNECs G3. All synchronous distant metastases were hepatic oligometastases have only a few metastatic spot. The median number of metastatic lymph-node was 1 (mean (s.d.) number, 1.2 (1.3); range, 0–7).

### Comparison of clinicopathologic characteristics between WD-pNETs G3 and PD-pNECs G3

A comparison of clinicopathological characteristics between patients with WD-pNETs G3 and PD-pNECs G3 is summarized in Table 1. Thirty patients had WD-pNETs G3 and 27 patients had PD-pNECs G3. Regarding the 30 WD-pNETs G3, there were 2 functional tumors (1 insulinoma and 1 VIPoma); the numbers of stage I, stage II, stage III and stage IV were 9, 13, 0 and 8, respectively; the median Ki-67 and mitotic count were 25 and 16.5, respectively (range 1–50 and 2–60). Regarding to the 27 PD-pNECs G3, eight were small cell NECs, and 19 were large cell NECs; the numbers of stage I, stage II, stage III and stage IV were 2, 9, 9 and 7, respectively; the median Ki-67 and mitotic count were 50 and 26, respectively (range 5–95 and 10–79).

**Table 1:** The clinicopathological characteristics of patients with well differentiated pancreatic neuroendocrine tumors (WD-pNETs) G3 and poorly differentiated pancreatic neuroendocrine carcinomas (PD-pNECs) G3.

G3 pNENs	Well-moderately differentiated pancreatic neuroendocrine tumors (pNETs) (*N* = 30), *N*	Poorly differentiated pancreatic neuroendocrine carcinomas (pNECs) (*N* = 27), *N*	*P* value
Sex			0.146
Female	18	11	
Male	12	16	
Functional pNENs			0.492^#^
Yes	2	0	
No	28	27	
Location			0.380
Head of the pancreas	14	9	
Distal pancreas	15	15	
Diffusion/retroperitoneum	1	3	
AJCC TNM stage			0.003
I	9	2	
II	13	9	
III	0	9	
IV	8	7	
Regional lymph nodes metastasis			0.005
Yes	11	20	
No	19	7	
Synchronous distant metastases			0.804
Yes	8	8	
No	22	19	
Initial treatments			0.091^#^
Initial surgical resection	29	22	
Initial chemotherapy	1	5	
Perineural invasion			0.599
Yes	13	14	
No	16	13	
Vascular invasion			0.046
Yes	8	14	
No	22	13	
Nonischemic tumor necrosis			0.259
Yes	7	10	
No	23	17	
Adjacent organs invasion			0.734
Yes	12	12	
No	18	15	
Primary multifocal tumors			1.000^#^
Yes	2	1	
No	28	26	
Ki-67^†^			0.001
Median, (range), %	25 (1–50)	50 (5–95)	
Mitotic count^†^			0.007
Median, (range)	16.5 (2–60)	26 (10–79)	
Tumor size^†^			0.637
Median, (range), cm	3.8 (1.9–17.0)	3.5 (1.8–16.0)	
Age^†^			0.961
Median, (range), year	54.5 (27–76)	54 (21–82)	
Serum CgA^†^			0.048
Median, (range), μg/L	74.9 (33.4–870)	58.3 (43.5–189.4)	
Serum NSE^†^			0.002
Median, (range), μg/L	13.8 (11.5–30)	27.6 (15.9–67.9)	

^†^Continuous variant; ^#^Fisher’s Exact Test.

**Figure 1:**
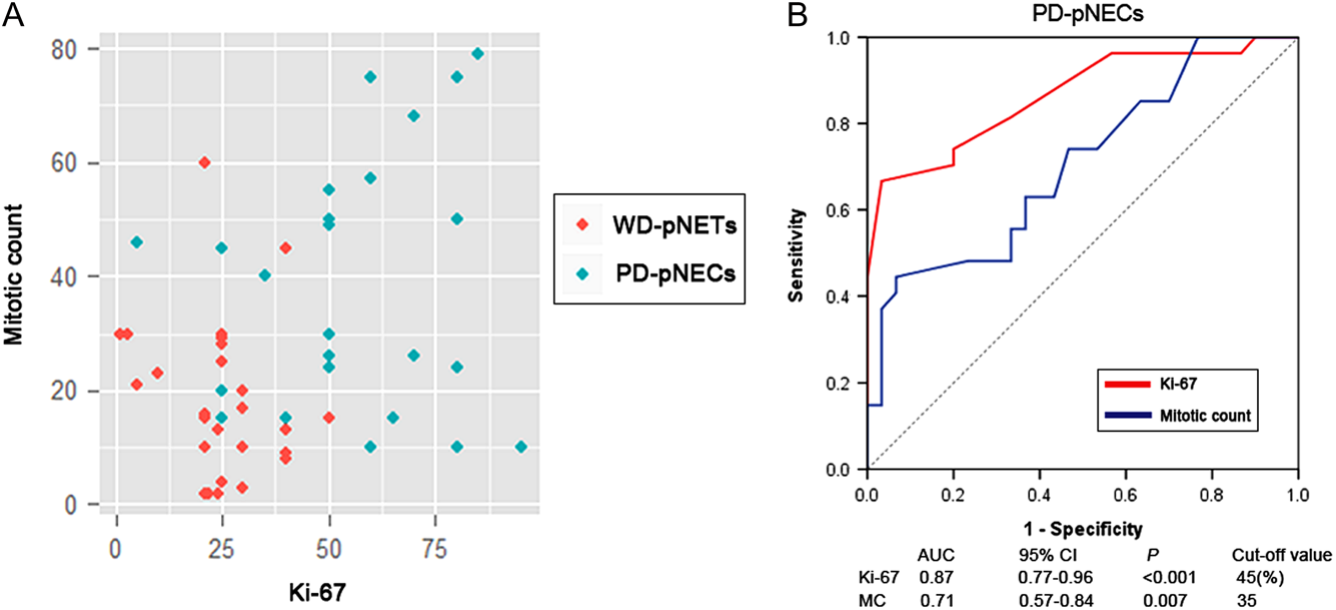
Diagnostic values of Ki-67 and mitotic count for different G3 pNENs. (A) The distributions of Ki-67 and mitotic count in patients with PD-pNECs G3 or WD-pNETs G3 showed remarkable disparitie. (B) Diagnostic value of Ki-67 and mitotic count for different G3 pNENs, a ROC graph was obtained to discriminate PD-pNECs from G3 pNENs; for Ki-67 and mitotic count, the area under the curve were 0.87 and 0.71, and cut-off value were 45 and 35, respectively.

The distributions of Ki-67 and mitotic count in patients with PD-pNECs or WD-pNETs showed remarkable disparities (Fig. 1A). To discriminate PD-pNECs from G3 pNENs, our ROC indicated that the cut-off values of Ki-67 index and mitotic count were 45 and 35, and the areas under the curve were 0.87 and 0.71, respectively (Fig. 1B). Considering clinical features, PD-pNECs were more common in patients with elevated Ki-67 index, elevated mitotic count, advanced AJCC TNM stage, vascular invasion and regional lymph-node metastases compared with WD-pNETs (*P* < 0.05). Interestingly, PD-pNECs were more likely to have elevated serum NSE levels and decreased serum CgA levels (*P* < 0.05). Finally, patients with perineural invasion or nonischemic tumor necrosis were associated with an increased frequency in PD-pNECs, although no statistically significant difference was found (*P* > 0.05).

### Therapeutic interventions

Among the entire G3 cohort, fifty-one patients initially underwent potentially curative resection of primary pNENs with lymph nodes dissection. Initial surgical treatments in combination with other therapies were as follows: eight patients had received hepatectomy, fourteen patients had received hepatic arterial chemoembolization (HACE), four patients had received radiofrequency ablation (RFA), five patients had received Octreotide LAR, three patients had received sunitinib, one patient had received everolimus, seven patients had received chemotherapy and four patients had received radiotherapy. In another subgroup of patients with initial chemotherapy, six biopsy-proven patients initially underwent chemotherapy (which included cisplatinum/etoposide or platinum/oxaliplatin-based regimens). Initial chemotherapies in combination with other therapies were as follows: one patient had received HACE, one patient had received RFA, one patient had received everolimus and one patient had received radiotherapy.

### Survival analysis and prognostic factors of G3 pNENs

**Figure 2:**
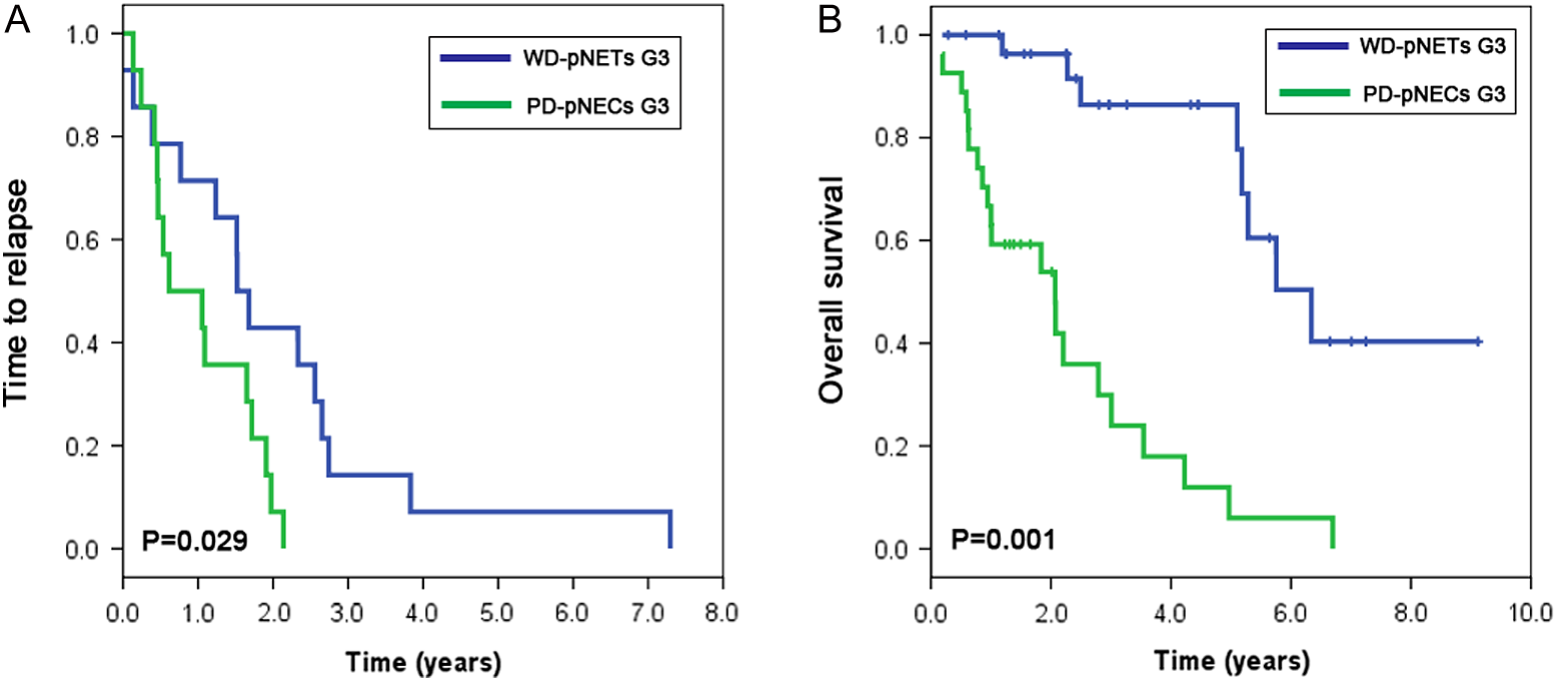
Survival analysis of different G3 pNENs. (A) The comparison of TTR between PD-pNECs G3 and WD-pNETs G3 by Kaplan–Meier analysis. (B) The comparison of OS between PD-pNECs G3 and WD-pNETs G3 by Kaplan–Meier analysis.

Among those with complete tumor remission obtained, the relapse rate was 32/49 and the median TTR duration for the total population was 1.37 years (mean relapse time 1.54 ± 1.48 years, range 0.13–7.3 years). Regarding to TTR, The association between 2017 WHO G3 grade and TTR duration was statistically significant, PD-pNECs could reduce TTR compared with WD-pNETs (*P* = 0.029, median (95% CI): 0.61 (0–1.56) vs 1.52 (1.22–1.82) years, HR (95% CI): 2.6 (1.1–6.3), Fig. 2A).

At the last follow-up, twenty-nine patients died from tumor recurrence or recurrence-related complications. The 1-, 3- and 5-year OS rates were respectively 84, 60 and 46%. In univariate analysis, OS rates were associated with morphologic differentiation (WD-pNETs vs PD-pNECs), Ki-67 index, AJCC TNM staging, synchronous distant metastases, initial treatments, vascular invasion, regional lymph nodes metastases, mitotic count and age (*P* < 0.05, Table 2). The 5-year OS of patients with PD-pNECs and WD-pNETs were 6% and 86%, and the median OS were 2.07 and 6.34 years (95% CI: (0.80–3.34) and (4.83–7.85)), respectively (Fig. 2B). In a subgroup analysis, small and large cell morphology is not a prognostic factor for PD-pNECs (*P* > 0.05). In addition, the median OS of those patients underwent initial surgical treatments and those underwent initial chemotherapy were 5.10 and 0.63 years (95% CI: (3.91–6.30) and (0.55–0.70)), respectively. Multivariate analyses illustrated that Ki-67 index, differentiation, AJCC TNM staging and vascular invasion were independent risk factors for OS (*P* < 0.05, Table 2).

**Table 2:** The clinicopathological characteristics in patients with G3 pNENs: univariate and multivariate survival analyses.

Variable	*N*	5-year OS (%)	Univariate *P* value	Multivariate *P* value	Multivariate HR for OS (95% CI)
Sex			0.176		
Female	29	61			
Male	28	30			
Functional pNENs			0.967		
Yes	2	NA			
No	55	46			
Differentiation			<0.001	0.006	4.0 (1.2–18.3)
WD-pNETs	30	86			
PD-pNECs	27	6			
Location			0.174		
Head of the pancreas	23	60			
Distal pancreas	30	38			
Diffusion/retroperitoneum	4	25			
AJCC TNM stage			<0.001	0.022	1.4 (0.6–3.5)
I	11	100			
II	22	57			
III	9	NA			
IV	15	21			
Regional lymph nodes metastasis			0.001	NS	2.0 (0.8–5.7)
Yes	31	21			
No	26	70			
Synchronous distant metastases			0.001	NS	1.5 (0.6–8.3)
Yes	16	19			
No	41	58			
Initial treatments			0.001	NS	0.2 (0.1–1.4)
Initial surgical resection	51	53			
Initial chemotherapy	6	NA			
Perineural invasion			0.509		
Yes	27	39			
No	29	61			
Vascular invasion			<0.001	0.008	4.3 (1.5–12.8)
Yes	22	11			
No	35	65			
Nonischemic tumor necrosis			0.385		
Yes	17	35			
No	40	59			
Adjacent organs invasion			0.842		
Yes	24	42			
No	33	49			
Primary multifocal tumors			0.654		
Yes	3	67			
No	54	45			
Ki-67^†^			<0.001	0.002	1.1 (1.0–1.1)
Median	30				
Range	1–95				
Mitotic count^†^			0.001	NS	1.0 (0.9–1.1)
Median	23				
Range	2–79				
Tumor size (cm)^†^			0.272		
Median	3.6				
Range	1.8–17				
Age^†^			0.010	NS	1.1 (1.0–1.1)
Median	54				
Range	21–82				

^†^Continuous variant.

NA, not applicable; NS, not significant.

## Discussion

The pNENs with high-grade (WHO G3) are generally heterogeneous malignant neoplasms showing a complex pattern of phenotypes. It presents a wide spectrum of clinical features that varies from individuals. WD-pNETs, although mostly presenting low to intermediate grade, could uncommonly contain regions with high proliferation that place them in the WHO G3 category (17). The etiology of WD-pNETs is genetically different from that of PD-pNECs. Several studies have reported genetic alterations in TP53 and RB1 of PD-pNECs (7). In contrast, the bulk of the evidence suggests that the ATRX and DAXX, and MEN-1genes are significantly mutated in most patients with highly proliferative G3 WD-pNETs, as are genes encoding key molecules of the mTOR signaling pathway (18, 19), and these genetic alterations are similar to those observed in the less malignant G1 and G2 WD-pNETs. It seems unlikely that PD-pNECs arise via progression from WD-pNETs with any frequency.

In our study, PD-pNECs were more prone to have elevated Ki-67 and mitotic count, advanced AJCC TNM stage, vascular invasion, regional lymph-node metastases compared with WD-pNETs, implying that PD-pNECs G3 present malignant biological behaviors from various aspects. Also, the distributions of Ki-67 and mitotic count in patients with PD-pNECs or WD-pNETs showed remarkable disparities. Consistently, another study indicated PD-pNECs were more common in patients with higher Ki-67 index, regional lymph-node metastases compared with WD-pNETs (20). Our study showed most G3 WD-pNETs patients had markedly high CgA levels, unlike G3 PD-pNECs patients (*P* < 0.05). Conversely, G3 PD-pNECs rarely showed increased CgA levels, they had elevated NSE levels (*P* < 0.05). It appears that WD-pNETs have a neuroendocrine cell lineage (21), and the loss of CgA expression in PD-pNECs indicates their partial neuroendocrine differentiation, in accord with the ‘on/off’ switch function of the *CHGA/CgA* gene, which alone is sufficient to drive neuroendocrine differentiation in mammalian cells (22). These clinical findings may be important as both WD-pNETs G3 and PD-pNECs G3 are strikingly biologically heterogeneous. Hence, WD-pNETs G3 should be differentiated from PD-pNECs by 2017 WHO grading. Mounting evidence indicated that Ki-67 index functions more effectively than mitotic count (11, 18), and our study reported Ki-67 index was more efficient in grading G3 pNENs. The ROC demonstrated that the diagnostic cut-off value of Ki-67 index was 45 (AUC: 0.87) in identifying PD-pNECs G3 from WD-pNETs G3, whereas mitotic count was 35 (AUC: 0.71). However, in our cohort, one WD-pNET case showed very low Ki-67 (2%) with high MC (>20%), and one PD-pNEC case showed low Ki-67 (5%) with high MC (>20%); this unusual phenomenon indicates Ki-67 algorithm cannot capture the full complexity of pNENs, a further improvement of the classifications can be expected by the inclusion of genetic differences.

The majority of patients were nonfunctional G3 pNENs at presentation, and more importantly, most commonly presented with relatively high regional lymph-node metastases rate. Thus, we recommended initial radical surgery with lymph-node dissection to achieve primary R0/R1 resection for potentially resectable G3 pNENs. In survival analysis, our cohort demonstrated initial surgical treatments seemed to achieve a better OS than initial chemotherapy (*P* < 0.05, HR = 0.2, 95% CI: 0.1–1.4). Similar results were found in nonmetastatic NET G3, surgery appeared to be the first option, and chemotherapy regimen should be in line with that implemented for neuroendocrine carcinoma when Ki-67 is above 55% (23). Surgical resection of localized nonmetastatic high-grade pNECs has also been associated with a relatively high 5-year survival of 43% (24). Clinically, the introduction of 2017 WHO G3 WD-pNET category is important because these usually show a poor response to first-line platinum-based chemotherapy which pNECs receive, while they respond favorably to surgery for resectable disease and to somatostatin analogs, everolimus and sunitinib in metastatic disease. Hence, surgical procedure seems to be the first option for potentially resectable G3 pNENs.

In the current study, the 5-year OS rate was 46% from initial baseline, which indicates that potentially resectable G3 pNENs have relatively ominous prognosis even long-term outcome could be reached in some cases when R0 resection were achieved. The relapse rates were 65.3% and the median TTR was 1.37 years, and PD-pNECs could significantly reduce TTR duration compared with WD-pNETs (*P* < 0.05). These evidences suggest postoperative G3 pNENs patients, especially the PD-pNECs, are at high risk for relapse and metachronous metastases after resection of the primary tumor. We examine dozens of clinicopathologic characteristics in greater detail and confirmed that morphologic differentiation (WD-pNETs vs PD-pNECs), Ki-67 index, AJCC TNM staging, synchronous distant metastases, initial treatments, vascular invasion, regional lymph nodes metastases, mitotic count and age emerged as independent predictors of OS. With respect to morphologic differentiation, well-differentiated lesions have characteristic organoid arrangements of the tumor cells, which are relatively uniform and produce abundant neurosecretory granules. Poorly differentiated lesions resemble less closely non-neoplastic neuroendocrine cells and have a more diffuse architecture and irregular nuclei and less cytoplasmic granularity (25). Thus, it must be acknowledged that, without differentiation, classification of G3 pNENs based on proliferative activity alone, may fail to unmask the underlying pathologic basis of different neoplastic entities, highlighting the necessity of 2017 WHO classification (15, 16).

Whether the small and large cell subtypes of PD-pNECs represent different subtypes or proliferative activities, is unclear so far. Our data showed survival was not different between these two subtypes. Evidence suggests that the presence of regional lymph-node metastases is independently correlated with decreased disease-specific survival in patients with high-grade pNECs (26). Regarding to synchronous distant metastases, current guidelines do not recommend surgery or debulking surgery for distant metastasis of high-grade pNECs, which can significantly reduce the prospects for long-term survival. However, some literature reported that surgery for metastatic high-grade pNENs may improve survival, with a 3-year survival of 69% among 12 patients (27). Another study demonstrated that aggressive locoregional treatment improves the outcome of liver metastases from G3 pNENs (28). Our data showed that comprehensive treatments of liver metastases including hepatectomy, hepatic arterial chemoembolization, RFA, octreotide, sunitinib, everolimus, chemotherapy, radiotherapy would improve clinical outcome with a 5-year OS of 46%. Notably, the vascular invasion has been observed to be an important factor that affected outcome. Similar results were found that vascular invasion is an independent predictor in pNETs and pNECs (29, 30). This may be explained by the tumor biology that G3 pNENs was a highly angiogenic neoplasm, dependent upon activated VEGFR2 signaling pathway (31).

As stated above, our results indicated that WD-pNETs can achieve a proliferative rate within G3 grade complicates the prognostic stratification of heterogeneous pNENs and suggested that introduction of 2017 WHO classification would be necessary. The morphologic differentiation seems to have been fully as important as proliferative activity. More detailed genetic inheritances are needed to unmask subtle differences between PD-pNECs G3 and WD-pNETs G3 in the future.

## Declaration of interest

The authors declare that there is no conflict of interest that could be perceived as prejudicing the impartiality of the research reported.

## Funding

This study was supported by National Natural Science Foundation of China (81401923, 81572294, 81702304, 81773068, 81702964); CSCO-Novartis neuroendocrine tumor development fund (2013); Novartis China GEP/NET Registry (CSMS955ACN11).
